# Supplementary material for: Pharmacodynamic Profiling of Amoxicillin: Targeting Multidrug-Resistant Gram-Positive Pathogens Staphylococcus aureus and Staphylococcus pseudintermedius in Canine Clinical Isolates
Source: Antibiotics (Basel). 2025 Jan 16;14(1):99. doi: 10.3390/antibiotics14010099 (PMC11761891; doi:10.3390/antibiotics14010099)
Supplement: Supplementary file 1 [file antibiotics-14-00099-s001.zip › antibiotics-3357646-supplementary.pdf]

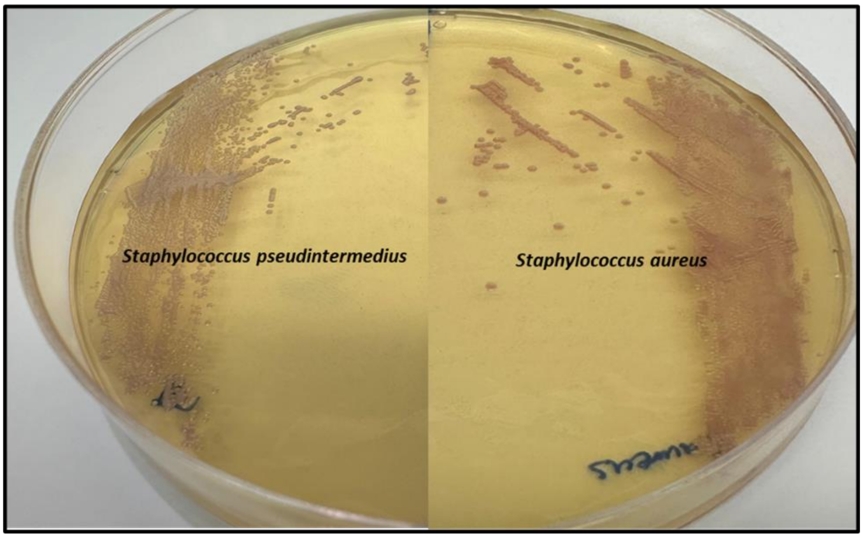

Figure S1: Bacterial colony of SA and SP.

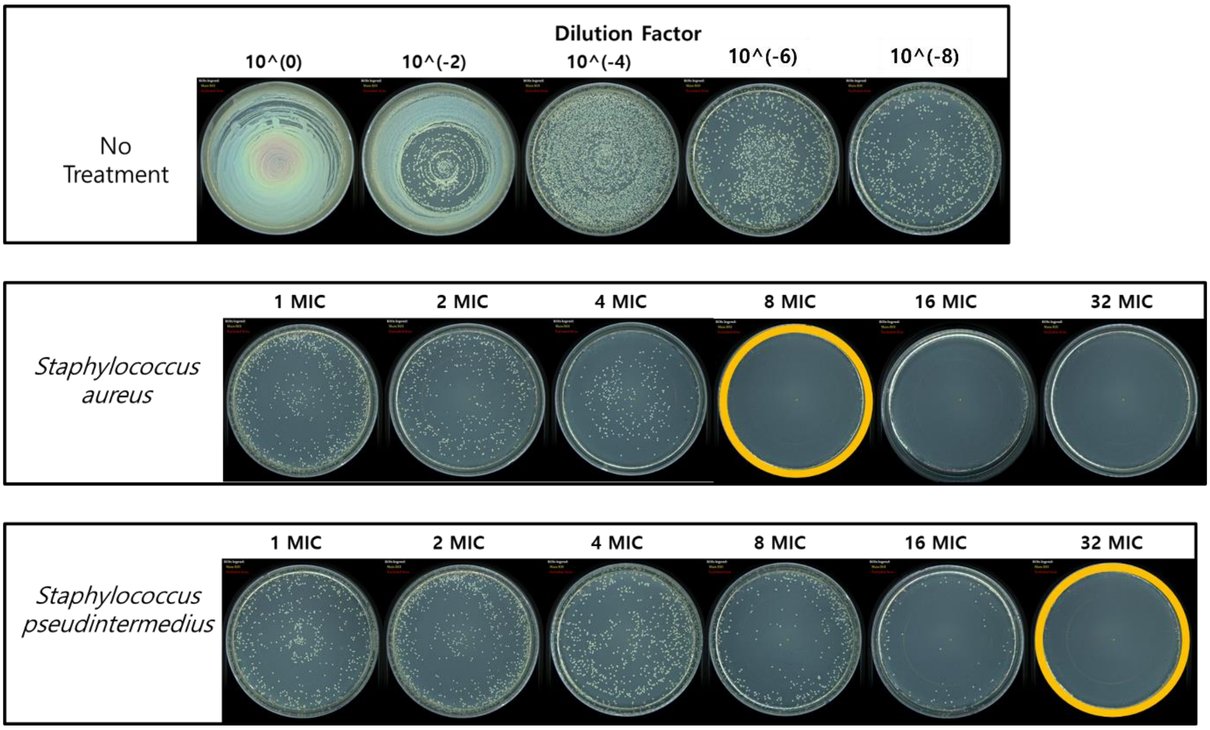

Figure S2: Bacterial growth and inhibition in Amx coated MPC agar plate.
